# Supplementary material for: The interactome of intact mitochondria by cross-linking mass spectrometry provides evidence for coexisting respiratory supercomplexes
Source: Mol Cell Proteomics. 2017 Dec 8;17(2):216–32. doi: 10.1074/mcp.RA117.000470 (PMC5795388; doi:10.1074/mcp.RA117.000470)
Supplement: Supplemental Data [file supp_RA117.000470_133922_0_supp_23978_fzffwf.zip › spectra_annotation/mito_DR_spectra_annotation/398-1-11-1-5-1.pdf]

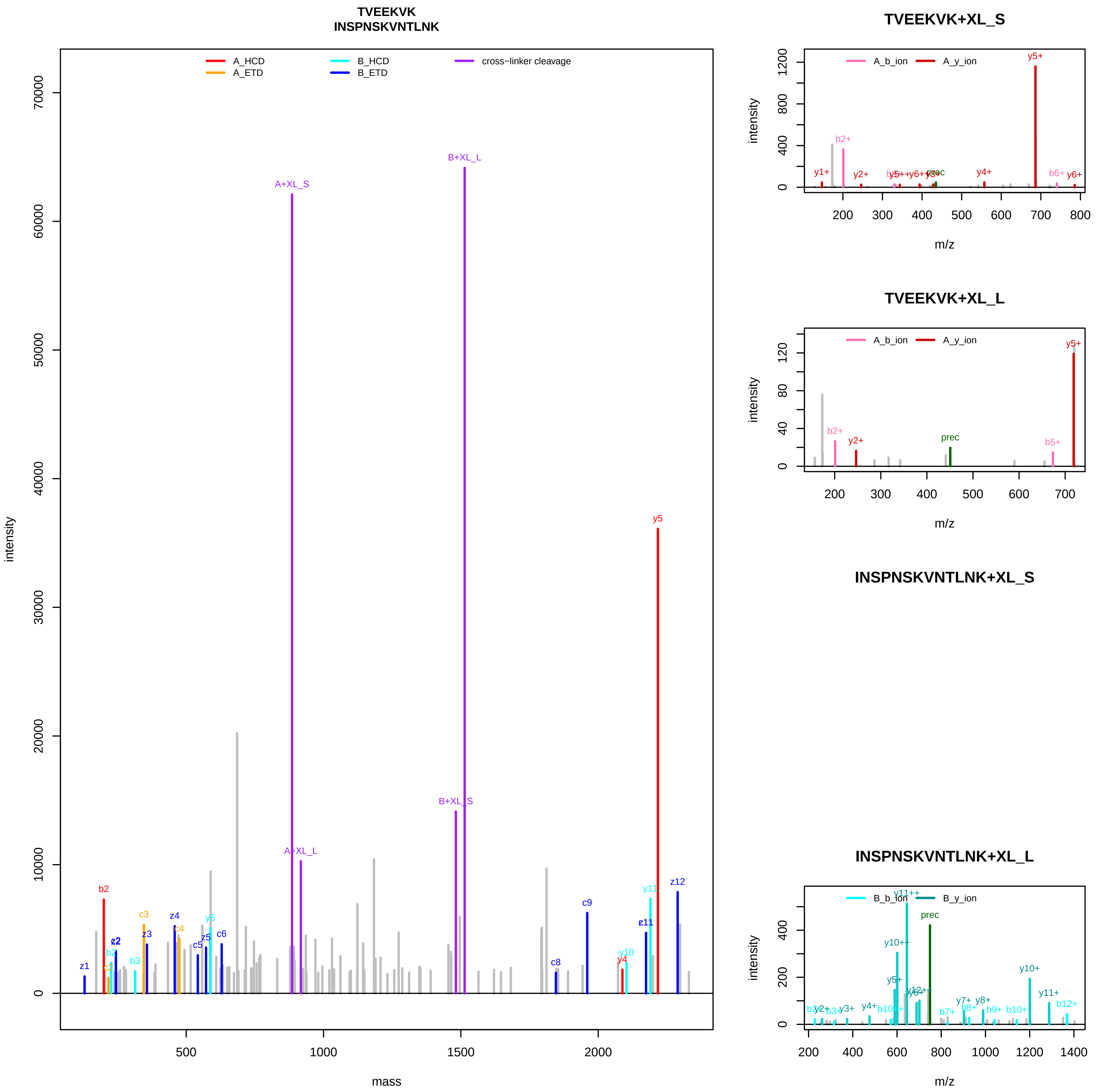

TVEEKVK
INSPNSKVNTLNK
A_HCD
A_ETD
B_HCD
B_ETD
cross−linker cleavage
intensity
mass
A+XL_S
B+XL_L
B+XL_S
A+XL_L
y5
b2
c3
z4
c4
y5
c5
z5
c6
z3
z2
c2
b2
b3
z1
c8
c9
y4
y10
z11
y11
z12
0
10000
20000
30000
40000
50000
60000
70000
500
1000
1500
2000
TVEEKVK+XL_S
A_b_ion
A_y_ion
y5+
b2+
y1+
y2+
prec
y4+
b6+
y6+
m/z
TVEEKVK+XL_L
b2+
y2+
prec
b5+
INSPNSKVNTLNK+XL_S
INSPNSKVNTLNK+XL_L
B_b_ion
B_y_ion
y11++
prec
y10++
y10+
y11+
b12+
y5+
y12++
y6+
y7+
y8+
b7+
b8+
b9+
b10+
y3+
y4+
